# Multi-Target Tracking Using an Improved Gaussian Mixture CPHD Filter

**DOI:** 10.3390/s16111964

**Published:** 2016-11-23

**Authors:** Weijian Si, Liwei Wang, Zhiyu Qu

**Affiliations:** College of Information and Communication Engineering, Harbin Engineering University, Harbin 150001, China; siweijian@hrbeu.edu.cn (W.S.); wang080006@hrbeu.edu.cn (L.W.)

**Keywords:** GM-CPHD filter, multi-target tracking, spooky effect, weight redistribution

## Abstract

The cardinalized probability hypothesis density (CPHD) filter is an alternative approximation to the full multi-target Bayesian filter for tracking multiple targets. However, although the joint propagation of the posterior intensity and cardinality distribution in its recursion allows more reliable estimates of the target number than the PHD filter, the CPHD filter suffers from the spooky effect where there exists arbitrary PHD mass shifting in the presence of missed detections. To address this issue in the Gaussian mixture (GM) implementation of the CPHD filter, this paper presents an improved GM-CPHD filter, which incorporates a weight redistribution scheme into the filtering process to modify the updated weights of the Gaussian components when missed detections occur. In addition, an efficient gating strategy that can adaptively adjust the gate sizes according to the number of missed detections of each Gaussian component is also presented to further improve the computational efficiency of the proposed filter. Simulation results demonstrate that the proposed method offers favorable performance in terms of both estimation accuracy and robustness to clutter and detection uncertainty over the existing methods.

## 1. Introduction

Multiple targets tracking (MTT) is a key technology for many practical applications in both military and civil fields [1,2]. In most cases, the MTT algorithm need to jointly estimate the time-varying number of targets and their individual states via using the measurements corrupted by noise and clutter. The most popular approaches, such as multiple hypothesis tracking (MHT) [3] and the joint probabilistic data association (JPDA) filter [4], are involved in traditional MTT algorithms to solve the problem of measurement origin uncertainty (also referred to as the data association problem). Generally, the association-based techniques suffer from heavy computational costs and can be very unreliable in the presence of detection uncertainty and clutter [5]. Alternatively, the MTT problem has been recast in the Bayesian filtering framework by modeling the multi-target systems using random finite set (RFS) formulation [6], and the resulting optimal multi-target Bayesian filter has laid the foundation for developing many innovative multi-target filters [7,8,9]. Based on moment approximation, the probability hypothesis density (PHD) filter [7] and cardinalized PHD (CPHD) filter [8] were proposed. Specifically, the PHD filter propagates the posterior intensity of the multi-target state, while the CPHD filter additionally propagates the cardinality distribution, i.e., the probability distribution of the number of targets. To implement the PHD and CPHD filters, the sequential Monte Carlo (SMC) method and the Gaussian mixture (GM) method have been introduced in [10,11,12]. During the past decade, the PHD and CPHD filters have been applied to many practical problems and generated substantial interest [13,14,15].

The main advantages of the PHD and CPHD filters over traditional methods are that they operate on the single-target state space and avoid the intractable problem of data association. In addition, they provide the ability to resolve the uncertainties in both the number of targets and their corresponding states in a cluttered environment. Compared with the PHD filter, the CPHD filter can achieve significant improvements in the accuracy of cardinality estimation and tracking performance [12,13]. Meanwhile, the GM-CPHD filter provides a closed form solution to the CPHD recursion, which makes it more computationally efficient than the SMC implementation. However, the studies in [16,17] demonstrated that the CPHD filter exhibits a counter-intuitive behavior: upon missed detections, the PHD mass of the undetected targets will be shifted to that of the detected targets, regardless of the spatial locations of the targets. This phenomenon is also referred to as the spooky effect [17] in the CPHD filter, and the amount of the shifted PHD mass depends on the total target number. One possible way to reduce this effect is to find a general decomposition of the CPHD filter with respect to separated regions and then apply the filter to each of the regions individually [16]. Unfortunately, no rigorous method has been reported to achieve this task for more general situations. To alleviate the PHD interaction via missed detections, a dynamic reweighting method was proposed in [18], where the components with large updated weights were exploited to compensate the components with small updated weights. However, this process suffers from the drawbacks that only the single-frame information is considered, and the total excess weight will be unreasonably assigned to all of the existing Gaussian components with lower updated weights, including the invalid birth components and the residual components, which belong to the detected targets, but are not merged into the corresponding detection components. Although many new techniques have been incorporated into the CPHD filter to improve its performance and generality [19,20,21], the spooky effect remains an inherently unfavorable factor for practical applications of the CPHD-based filter; because the exaggerated reduction of weights on tracks with missed detections might significantly degrade tracking performance and even lose track of targets [18,20].

Another critical issue for the CPHD filter is the high computational complexity arising from the joint propagation of the intensity function and cardinality distribution, which directly depends on the number of measurements with a cubic relationship [12]. Therefore, when there is a large number of false alarms or clutters in the monitoring region, the real-time performance of the algorithm would be worse. At present, the existing solutions mainly resort to gating techniques [22,23] to improve the computational efficiency of the CPHD filter. The adaptive gating method outlined in [23] shows some advantages as compared with the standard elliptical gating method. However, the adaptive characteristic is obtained by directly using the predicted weight of each component to enlarge the gate sizes, which will result in an excessive increase in the validation region for the component with a large weight. As a result, more non-target-originated measurements may be selected for the filter. This goes against the principle of reducing the number of candidate measurements and results in low overall effectiveness.

In this paper, we propose an improved GM-CPHD filter, which aims at addressing the aforementioned drawbacks of the original version. In particular, starting from distinguishing between the detected components and the undetected components, a weight redistribution scheme is introduced into the update step of the filter to compensate the updated weights of the undetected targets, where the information in multiple frames (missed detection or consecutive missed detections) is considered to redistribute the total transferred PHD mass to each undetected target. The resulting filter can effectively reduce the spooky effect in the GM-CPHD filter. Besides, a principled gating strategy is also proposed to improve the computational efficiency of the filter, which adaptively enlarges the gate sizes for undetected targets in the previous iteration to ensure the inclusion of true measurements. Simulation results demonstrate that the proposed method yields favorable tracking performance and good robustness in missed detection and a cluttered environment.

The remainder of this paper is organized as follows. Section 2 presents an overview of the RFS formulation of the MTT problem, together with the CPHD filter and its GM implementation. Section 3 presents the proposed method. Simulation results are presented in Section 4, and conclusions are drawn in Section 5.

## 2. Background

### 2.1. Random Finite Set Model

In MTT scenarios, the number of targets often changes over time. As a consequence, the unknown multi-target state generates corresponding measurements whose number is also time-varying at each time step. The random finite set (RFS) approach provides a mathematically-elegant treatment of multi-target systems by modeling the collections of target states and measurements as RFSs. For example, if there are $n_{k}$ targets with states $x_{k,1},x_{k,2},\ldots ,x_{k,n_{k}}$ and $m_{k}$ measurements $z_{k,1},z_{k,2},\ldots ,z_{k,m_{k}}$ at time k, then the RFS representation of the multi-target state and measurements are respectively defined as [7,11]:

(1) $$X_{k}=\{x_{k,1},x_{k,2},\ldots ,x_{k,n_{k}}\}\in ℱ(X)$$

(2) $$Z_{k}=\{z_{k,1},z_{k,2},\ldots ,z_{k,m_{k}}\}\in ℱ(Z)$$

where ℱ(X) and ℱ(Z) are the collections of all finite subsets of single-target state space and single-target observation space, respectively. In general, some clutter measurements may be collected, and some of the existing and newborn targets may not be detected due to the imperfect detectors. Given a multi-target state $X_{k-1}$ at time k−1, we consider the multi-target dynamics modeled by:

(3) $$X_{k}=\left[\underset{\zeta \in X_{k-1}}{\cup }S_{k|k-1}(\zeta )\right]\cup \Gamma_{k}$$

where $S_{k|k-1}(\zeta )$ denotes the RFS of survival target at time k that evolved from a target given the state ζ at the previous time step, and $\Gamma_{k}$ denotes the RFS of newborn targets at time k.

The received measurements by the sensor are modeled by:

(4) $$Z_{k}=\left[\underset{x\in X_{k}}{\cup }\Theta_{k}(x)\right]\cup K_{k}$$

where $\Theta_{k}(x)$ denotes the RFS of measurement, which originates from the true target and $K_{k}$ denotes the RFS of clutter measurements (or false alarms) at time k. Based on the RFS model and finite set statistics (FISST) theory, the optimal multi-target Bayesian filter was developed to propagate the posterior density of the multi-target state recursively in time; further details on mathematical derivations and analysis can be found in [6].

### 2.2. CPHD Filter and Its GM Implementation

The CPHD filter can be regarded as a higher-order generalization of the PHD filter, which still remains the first-order PHD of the multi-target state, but the higher-order information in target number [8]. Concretely, the CPHD filter jointly propagates the intensity function and the cardinality distribution to improve the overall estimation accuracy of target number. Let $v_{k-1}$ denote the posterior intensity and $p_{k-1}$ denote the posterior cardinality distribution at time k−1, then the prediction step of the CPHD filter is given by [12]:

(5) $$p_{k|k-1}(n)=\sum_{j=0}^{n}p_{\Gamma ,k}(n-j)\prod_{k|k-1}[v_{k-1},p_{k-1}](j)$$

(6) $$v_{k|k-1}(x)=\int p_{S,k}(\zeta )f_{k|k-1}(x|\zeta )v_{k-1}(\zeta )d\zeta +\gamma_{k}(x)$$

where $p_{\Gamma ,k}(\cdot )$ is the cardinality distribution of birth targets at time k, $\gamma_{k}(x)$ is the intensity of spontaneous births at time k, $p_{S,k}(\zeta )$ is the probability that a target will survive at time k given the state ζ at the previous time step, $f_{k|k-1}(\cdot |\zeta )$ is the transition probability density of a single target and:

(7) $$\prod_{k|k-1}\left[v,p\right](j)=\sum_{l=j}^{\infty }C_{j}^{l}\frac{{\langle p_{S,k},v\rangle }^{j}{\langle 1-p_{S,k},v\rangle }^{l-j}}{{\langle 1,v\rangle }^{l}}p(l)$$

with $C_{j}^{l}=\frac{l!}{j!(l-j)!}$ representing the binomial coefficient and 〈⋅,⋅〉 representing the inner product operation defined between two real-valued functions a and b by 〈a,b〉=∫a(x)b(x)dx.

Given the predicted intensity $v_{k|k-1}$ and the predicted cardinality distribution $p_{k|k-1}$, the update step of the CPHD filter is given by:

(8) $$p_{k}(n)=\frac{\Psi_{k}^{0}[v_{k|k-1},Z_{k}](n)p_{k|k-1}(n)}{\langle \Psi_{k}^{0}[v_{k|k-1},Z_{k}],p_{k|k-1}\rangle }$$

(9) $$\begin{array}{ll} v_{k}(x) & =\frac{\langle \Psi_{k}^{1}[v_{k|k-1},Z_{k}],p_{k|k-1}\rangle }{\langle \Psi_{k}^{0}[v_{k|k-1},Z_{k}],p_{k|k-1}\rangle }\times [1-p_{D,k}(x)]v_{k|k-1}(x)\\ & +\sum_{z\in Z_{k}}\frac{\langle \Psi_{k}^{1}[v_{k|k-1},Z_{k}\backslash \{z\}],p_{k|k-1}\rangle }{\langle \Psi_{k}^{0}[v_{k|k-1},Z_{k}],p_{k|k-1}\rangle }\times \psi_{k,z}(x)v_{k|k-1}(x) \end{array}$$

where:

(10) $${ϒ}_{k}^{u}[v,Z](n)=\sum_{j=0}^{\min(|Z|,n)}\left(|Z|-j)!p_{K,k}(|Z|-j\right)P_{j+u}^{n}\times \frac{{\langle 1-p_{D,k},v\rangle }^{n-(j+u)}}{{\langle 1,v\rangle }^{n}}e_{j}(\Xi_{k}(v,Z))$$

(11) $$\psi_{k,z}(x)=\frac{\langle 1,\kappa_{k}\rangle }{\kappa_{k}(z)}g_{k}(z|x)p_{D,k}(x)$$

(12) $$\Xi_{k}(v,Z)=\{\langle v,\psi_{k,z}\rangle :z\in Z\}$$

$p_{K,k}(\cdot )$ is the cardinality distribution of clutter at time k; $p_{D,k}(x)$ is the probability of detection for a target in state x; $g_{k}(z|\cdot )$ is the measurement likelihood of individual targets; $\kappa_{k}(z)$ is the intensity of clutter measurements; $P_{j}^{n}=\frac{n!}{(n-j)!}$; and $e_{j}(\cdot )$ is the elementary symmetric function (see [12] for more details).

It can be observed that the CPHD filter still involves multiple integrals in its recursion and admits no closed-form solution in general, except the class of linear Gaussian multi-target systems [11,12]. For the linear Gaussian multi-target models, the dynamical model and measurement model of individual targets are required to follow:

(13) $$f_{k|k-1}(x|\zeta )=N(x;F_{k-1}\zeta ,Q_{k-1})$$

(14) $$g_{k}(z|x)=N(z;H_{k}x,R_{k})$$

where N(⋅;m,P) denotes a standard Gaussian density with mean m and covariance P, $F_{k-1}$ denotes the state transition matrix, $H_{k}$ denotes the observation and $Q_{k-1}$ and $R_{k}$ denote the covariance of process noise and observation noise, respectively. Moreover, assuming that the survival probability and detection probability of each target are state independent and the intensity of birth RFS can be modeled as:

(15) $$\gamma_{k}(x)=\sum_{j=1}^{J_{\gamma ,k}}w_{\gamma ,k}^{j}N(x;m_{\gamma ,k}^{j},P_{\gamma ,k}^{j})$$

where the weight $w_{\gamma ,k}^{j}$, mean $m_{\gamma ,k}^{j}$ and covariance $P_{\gamma ,k}^{j}$ are the given model parameters. The following prediction and update steps [12] show how the CPHD filter analytically propagates the multi-target posterior intensity and the cardinality distribution in time based on the CPHD recursion Equations (5)–(9).

Prediction: Suppose at time k−1, given the posterior cardinality distribution $p_{k}$ and the GM representation of $v_{k}$ as:

(16) $$v_{k-1}(x)=\sum_{i=1}^{J_{k-1}}w_{k-1}^{i}N(x;m_{k-1}^{i},P_{k-1}^{i})$$

The CPHD prediction is given by:

(17) $$p_{k|k-1}(n)=\sum_{j=0}^{n}p_{\Gamma ,k}(n-j)\sum_{l=j}^{\infty }C_{j}^{l}p_{k-1}(l)p_{S,k}^{j}{\left(1-p_{S,k}\right)}^{l-j}$$

(18) $$v_{k|k-1}(x)=p_{S,k}\sum_{j=1}^{J_{k-1}}w_{k-1}^{j}N(x;m_{S,k|k-1}^{j},P_{S,k|k-1}^{j})+\gamma_{k}(x)$$

where $\gamma_{k}(x)$ is given in Equation (15), and:

(19) $$m_{S,k|k-1}^{j}=F_{k-1}m_{k-1}^{j}$$

(20) $$P_{S,k|k-1}^{j}=F_{k-1}P_{k-1}^{j}F_{k-1}^{T}+Q_{k-1}$$

After the prediction step, $v_{k|k-1}$ can be rewritten as:

(21) $$v_{k|k-1}(x)=\sum_{i=1}^{J_{k|k-1}}w_{k|k-1}^{i}N(x;m_{k|k-1}^{i},P_{k|k-1}^{i})$$

Update: Given the predicted $p_{k|k-1}$ and $v_{k|k-1}$, the CPHD update is given by:

(22) $$p_{k}(n)=\frac{{ϒ}_{k}^{0}[w_{k|k-1},Z_{k}](n)p_{k|k-1}(n)}{\langle {ϒ}_{k}^{0}[w_{k|k-1},Z_{k}],p_{k|k-1}\rangle }$$

(23) $$v_{k}(x)=\frac{\langle {ϒ}_{k}^{1}[w_{k|k-1},Z_{k}],p_{k|k-1}\rangle }{\langle {ϒ}_{k}^{0}[w_{k|k-1},Z_{k}],p_{k|k-1}\rangle }\times [1-p_{D,k}]v_{k|k-1}(x)+\sum_{z\in Z_{k}}\sum_{j=1}^{J_{k|k-1}}w_{k}^{j}(z)N(x;m_{k}^{j}(z),P_{k}^{j})$$

where:

(24) $${ϒ}_{k}^{u}[w,Z](n)=\sum_{j=0}^{\min(|Z|,n)}\left(|Z|-j)!p_{K,k}(|Z|-j\right)P_{j+u}^{n}\times \frac{{\left(1-p_{D,k}\right)}^{n-(j+u)}}{{\langle 1,w\rangle }^{j+u}}e_{j}(\Lambda_{k}(w,Z))$$

(25) $$\Lambda_{k}(w,Z)=\left\{\frac{\langle 1,\kappa_{k}\rangle }{\kappa_{k}(z)}p_{D,k}w^{T}q_{k}(z):z\in Z\right\}$$

(26) $$w_{k|k-1}={\left[w_{k|k-1}^{1},\cdots ,w_{k|k-1}^{J_{k|k-1}}\right]}^{T}$$

(27) $$q_{k}(z)={\left[q_{k}^{1}(z),\cdots ,q_{k}^{J_{k|k-1}}(z)\right]}^{T}$$

(28) $$q_{k}^{j}(z)=N(z;\eta_{k|k-1}^{j},S_{k|k-1}^{j})$$

(29) $$\eta_{k|k-1}^{j}=H_{k}m_{k|k-1}^{j}$$

(30) $$S_{k|k-1}^{j}=H_{k}P_{k|k-1}^{i}H_{k}^{T}+R_{k}$$

(31) $$w_{k}^{j}(z)=p_{D,k}w_{k|k-1}^{j}q_{k}^{j}(z)\times \frac{\langle {ϒ}_{k}^{1}[w_{k|k-1},Z_{k}\backslash \{z\}],p_{k|k-1}\rangle }{\langle {ϒ}_{k}^{0}[w_{k|k-1},Z_{k}],p_{k|k-1}\rangle }\frac{\langle 1,\kappa_{k}\rangle }{\kappa_{k}(z)}$$

(32) $$m_{k}^{j}(z)=m_{k|k-1}^{j}+K_{k}^{j}\left(z-\eta_{k|k-1}^{j}\right)$$

(33) $$P_{k}^{j}=\left[I-K_{k}^{j}H_{k}\right]P_{k|k-1}^{j}$$

(34) $$K_{k}^{j}=P_{k|k-1}^{j}H_{k}^{T}{\left[S_{k|k-1}^{j}\right]}^{-1}$$

More details on the mathematical derivation about the GM-CPHD filter can be found in [12]. In practice, the non-unity probability of detection of an MTT system leads to detection uncertainty. Although the CPHD filter has the ability to handle misdetections during its filtering iterations, the spooky effect is undesirable. For the GM-CPHD filter, the influence of the spooky effect is reflected in gaining or losing the weights of Gaussian components: the weights of the detected targets will increase, while the weights of the undetected targets will be artificially decreased in proportion to the estimated target number [17]. Thus, the filter is prone to report multiple estimates from detected targets in place of the undetected targets and even lose track of targets, which seriously deteriorates the estimation accuracy and tracking performance.

## 3. Improved GM-CPHD Filter

### 3.1. The Proposed GM-CPHD Filter

In the GM representation of the posterior intensity, many Gaussian components will be propagated and preserved at each time step [12]. Assuming that the posterior intensity $v_{k-1}$ is approximated by a GM of the same form as Equation (16) at time k−1, in order to distinguish between the confirmed Gaussian components (reported as state estimates) and the tentative components in ${\left\{w_{k-1}^{i},m_{k-1}^{i},P_{k-1}^{i}\right\}}_{i=1}^{J_{k-1}}$, we mark the parameter of each Gaussian component with a tag β, denoted as ${\left\{w_{k-1,\beta }^{i},m_{k-1,\beta }^{i},P_{k-1,\beta }^{i}\right\}}_{i=1}^{J_{k-1}}$, where:

(35) $$\beta =\left\{ \begin{array}{ll} 0 & \mathrm{for}\;\mathrm{others}\\ 1 & \mathrm{for}\;\mathrm{confirmed}\;\mathrm{components} \end{array}\right.$$

Note that the idea of using a tag or a label to serve as an indicator has been adopted in [21], wherein the newborn targets are distinguished from the existing targets to derive novel extensions of the PHD and CPHD filter. Our work, however, uses this technique to mark the parameter of each Gaussian component for the development of the subsequent weight redistribution scheme. Then, $v_{k-1}$ can be expressed as:

(36) $$v_{k-1}(x)=\sum_{\beta =0}^{1}\sum_{i=1}^{J_{k-1}}w_{k-1,\beta }^{i}N(x;m_{k-1,\beta }^{i},P_{k-1,\beta }^{i})$$

Besides, considering the possible misdetections for the existing targets, a counter variable $n_{\mathrm{miss}}$ is also introduced to record how many times the consecutive missed detection happens on each component. The value of $n_{\mathrm{miss}}$ for each component will be assigned during the filtering iterations.

Prediction: Since the parameters β and $n_{\mathrm{miss}}$ have no influence on the prediction of the posterior intensity and cardinality distribution, the prediction step is performed according to the original Equations (17) and (18). The components associated with the existing targets retain their tags and $n_{\mathrm{miss}}$ at this stage. Meanwhile, β=0 and $n_{\mathrm{miss}}=0$ are assigned to the components arising from $\gamma_{k}$ in Equation (15). Hence, let r represent the number of the confirmed components; the predicted intensity can be rewritten as:

(37) $$\begin{array}{ll} v_{k|k-1}(x) & =\sum_{\beta =0}^{1}\sum_{i=1}^{J_{k|k-1}}w_{k|k-1,\beta }^{i}N(x;m_{k|k-1,\beta }^{i},P_{k|k-1,\beta }^{i})\\ & =\sum_{i=1}^{r}w_{k|k-1,1}^{i}N(x;m_{k|k-1,1}^{i},P_{k|k-1,1}^{i})+\sum_{i=r+1}^{J_{k|k-1}}w_{k|k-1,0}^{i}N(x;m_{k|k-1,0}^{i},P_{k|k-1,0}^{i}) \end{array}$$

Update: Given the predicted intensity $v_{k|k-1}$ and predicted cardinality distribution $p_{k|k-1}$, the cardinality update is still computed according to the original Equation (22) without regard to the parameters β and $n_{\mathrm{miss}}$. However, for the intensity $v_{k|k-1}$, the update step is performed in a different way within the CPHD filtering scheme.

Let $v_{k|k-1}(x,1)$ denote the intensity associated with ${\left\{w_{k|k-1,1}^{i},m_{k|k-1,1}^{i},P_{k|k-1,1}^{i}\right\}}_{i=1}^{r}$ and $v_{k|k-1}(x,0)$ denote the intensity associated with ${\left\{w_{k|k-1,0}^{i},m_{k|k-1,0}^{i},P_{k|k-1,0}^{i}\right\}}_{i=1}^{J_{k|k-1}-r}$; we have $v_{k|k-1}(x)=v_{k|k-1}(x,1)+v_{k|k-1}(x,0)$. According to Equation (23), the updated posterior intensity $v_{k}$ consists of two parts: the missed detection update term and the detection update term. Based on the fact that the spooky effect is mainly caused by the improper update of the undetected targets, the following update calculations for $v_{k|k-1}(x,0)$ and $v_{k|k-1}(x,1)$ are proposed.

For the components in $v_{k|k-1}(x,0)$, they consist of both the tentative components with negligible weights and the birth components, which need to be confirmed. Based on the analysis in [18], the total transferred PHD mass on these components is relatively small, and therefore, we propose to update this part of intensity via Equation (23) as:

(38) $$v_{k}(x,0)=\Phi_{\mathrm{miss}}v_{k|k-1}(x,0)+\sum_{z\in Z_{k}}\sum_{j=1}^{J_{k|k-1}-r}w_{k,0}^{j}(z)N(x;m_{k,0}^{j}(z),P_{k,0}^{j})$$

where $w_{k,0}^{j}(z)$, $m_{k,0}^{j}(z)$ and $P_{k,0}^{j}$ are the corresponding updated parameters obtained according to Equations (31)–(33) for the components with β=0 and $\Phi_{\mathrm{miss}}$ is the updating factor for the missed detection update term and is defined as (for notational convenience):

(39) $$\Phi_{\mathrm{miss}}=\frac{\langle {ϒ}_{k}^{1}[w_{k|k-1},Z_{k}],p_{k|k-1}\rangle }{\langle {ϒ}_{k}^{0}[w_{k|k-1},Z_{k}],p_{k|k-1}\rangle }\times [1-p_{D,k}]$$

In addition, all of these updated components retain the tags of the underlying predicted components, and $n_{\mathrm{miss}}=0$ is assigned to them. Note that the term $w_{k|k-1}$ (defined by Equation (26)) in $\langle {ϒ}_{k}^{1}[w_{k|k-1},Z_{k}],p_{k|k-1}\rangle$ and $\langle {ϒ}_{k}^{0}[w_{k|k-1},Z_{k}],p_{k|k-1}\rangle$ of Equation (39) contains all weights ${\left\{w_{k|k-1,\beta }^{i}\right\}}_{i=1}^{J_{k|k-1}}$ with both β=0 and β=1; thus, we omit explicit reference to the tag β here.

For the components in $v_{k|k-1}(x,1)$, they contain all confirmed Gaussian components with high weights that are closely related to true targets [12]. When missed detections occur, the spooky effect mainly affects the weights of these components. To alleviate this problem, the proposed update process is implemented by introducing a weight redistribution scheme. First, we need to identify whether a component has the corresponding measurement in $Z_{k}$. A close inspection of Equation (23) reveals that each component in $v_{k|k-1}(x,1)$ gives rise to $\left(1+\left|Z_{k}\right|\right)$ terms in the updated mixture. Given a confirmed component characterized by $\left\{w_{k|k-1,1}^{i},m_{k|k-1,1}^{i},P_{k|k-1,1}^{i}\},i\in \{1,2,\ldots ,r\right\}$, and letting $m=1,\ldots ,m_{k}$ denote the index of a measurement in $Z_{k}$, we define the summation:

(40) $$W_{\mathrm{sum}}^{i}=\sum_{m=1}^{m_{k}}w_{k,1}^{i}(z_{k,m})$$

where $w_{k,1}^{i}(z_{k,m})$ represents the updated weight of the *i*-th component using measurement $z_{k,m}\in Z_{k}$. According to the updating principle of the GM-CPHD filter [12,16], a target-originated measurement will generate a significant weight for the corresponding Gaussian component representing the target. By contrast, clutter tends to generate zero to the updated weights of all components. Due to the unknown relations between targets and measurements in the CPHD filter, a principled threshold rule is exploited here, i.e., the component whose $W_{\mathrm{sum}}$ is greater than a threshold $T_{\det}$ is considered as a detected component and otherwise is deemed as an undetected component. Note that $T_{\det}$ is an empirical parameter for the practical application of our method. Considering the fact that $0\leq W_{\mathrm{sum}}^{i}\leq 1$ holds in general and there exist measurement error and clutter, the smaller the $T_{\det}$, the more detected components tend to be selected. Accordingly, some of these components tend to have negligible updated weights. Therefore, it is preferable to select the components that make certain contributions to the underlying targets with respect to the given measurements, and such components are more likely to be the detected components. To accomplish this, the range of this value is suggested to be 0.1–0.5, where a relative large value can be used for the tracking scenarios with high measurement accuracy, and vice versa.

Based on the results above, a weight redistribution method is proposed to compensate the updated weights of the undetected components. The pseudo-code of the proposed update process for $v_{k|k-1}(x,1)$ is presented in Algorithm 1, where the notation $z_{k,0}$ corresponds to the missed detection of a component. In fact, when missed detections occur, the true weight of an undetected target cannot be obtained in the CPHD recursion. As pointed out by [18], the missed detection update part of the PHD corresponding to detected components arises from the PHD of the undetected components. Thus, the basic idea behind our method is to determine the total PHD mass $W_{\mathrm{tr}}$ originating from the undetected components and then redistribute them back to the PHD regions of the undetected components (the updated components in $G_{\mathrm{miss}-\mathrm{up}}$ of Algorithm 1). Considering that the existence of detection uncertainty in multi-target environments may lead to missed detections or consecutive missed detections of multiple targets, in such cases, it is difficult to decide how much the PHD mass should be assigned to each undetected target. On the other hand, it is reasonable to consider the information in multiple frames because the deserved PHD of a target with consecutive missed detections should decrease as the increase of the number of consecutive missed detections. 

**Algorithm 1.** Pseudo-code for updating the confirmed Gaussian components (at time k>1)**Given**
${\left\{w_{k|k-1,1}^{i},m_{k|k-1,1}^{i},P_{k|k-1,1}^{i},n_{miss}^{i}\right\}}_{i=1}^{r}$, $Z_{k}$, the threshold $T_{\det}$ and the attenuation function $\alpha_{P}(n)$.**Step 0.** Set $G_{\mathrm{up}}=\{\}$, $G_{\mathrm{miss}-\mathrm{up}}=\{\}$ and $W_{\mathrm{tr}}=0$.**Step 1.** Update computation for detected components and undetected components.   **for**
i=1,…,r     **for**
$m=1,\ldots ,m_{k}$        Compute $w_{k,1}^{i}(z_{k,m})$ using (31).     end     Compute $W_{\mathrm{sum}}^{i}$ using (40).     **if**
$W_{\mathrm{sum}}^{i}\geq T_{\det}$        Compute $m_{k,1}^{i}(z_{k,m})$ and $P_{k,1}^{i}$ using (32)–(33).        $m^{*}=\arg\underset{m=1,\ldots ,m_{k}}{\max}\left(w_{k,1}^{i}(z_{k,m})\right)$.        Set $\beta^{i,m}=\left\{ \begin{array}{ll} 1 & m=m^{*}\\ 0 & \forall m=1,\ldots ,m_{k},m\neq m^{*} \end{array}\right.$.        Assign $n_{\mathrm{miss}}^{i,m}=0$ for $m=1,\ldots ,m_{k}$.        $\begin{array}{ll} G_{\mathrm{up}}= & G_{\mathrm{up}}\cup \{w_{k,1}^{i}(z_{k,m^{*}}),m_{k,1}^{i}(z_{k,m^{*}}),P_{k,1}^{i},n_{\mathrm{miss}}^{i,m^{*}}\}\\ & \cup {\left\{w_{k,0}^{i}(z_{k,t}),m_{k,0}^{i}(z_{k,t}),P_{k,0}^{i},n_{\mathrm{miss}}^{i,t}\right\}}_{t=1}^{m_{k}-1} \end{array}$.        $W_{\mathrm{tr}}=W_{\mathrm{tr}}+\Phi_{\mathrm{miss}}w_{k|k-1,1}^{i}$.     **else**        $n_{\mathrm{miss}}^{i}=n_{\mathrm{miss}}^{i}+1$.        $w_{k,1}^{i}(z_{k,0})=\Phi_{\mathrm{miss}}w_{k|k-1}^{i}$; $m_{k,1}^{i}(z_{k,0})=m_{k|k-1}^{i}$; $P_{k,1}^{i}=P_{k|k-1}^{i}$.        $G_{\mathrm{miss}-\mathrm{up}}=G_{\mathrm{miss}-\mathrm{up}}\cup \left\{w_{k,1}^{i}(z_{k,0}),m_{k,1}^{i}(z_{k,0}),P_{k,1}^{i},n_{\mathrm{miss}}^{i}\right\}$.     **end**   **end****Step 2.** Modify the updated weights of the Gaussian components in $G_{\mathrm{miss}-\mathrm{up}}$.     Compute $\alpha_{P}(n_{\mathrm{miss}}^{j^{\prime }})$ using (41) for all $j^{\prime }=1,\ldots ,d$ (d is the number of components in $G_{\mathrm{miss}-\mathrm{up}}$).Normalize all $\alpha_{P}(n_{\mathrm{miss}}^{j^{\prime }})$ by ${\tilde{\alpha }}_{P}(n_{\mathrm{miss}}^{j^{\prime }})=\frac{\alpha_{P}(n_{\mathrm{miss}}^{j^{\prime }})}{\sum_{j^{\prime }=1}^{d}\alpha_{P}(n_{\mathrm{miss}}^{j^{\prime }})}$.     **for**
$j^{\prime }=1,\ldots ,d$ Compensate the weights of the undetected component by:$w_{k,1}^{j^{\prime }}(z_{k,0})=w_{k,1}^{j^{\prime }}(z_{k,0})+{\tilde{\alpha }}_{P}(n_{\mathrm{miss}}^{j^{\prime }})W_{\mathrm{tr}}$     **end****Output:**
$G_{\mathrm{up}}=G_{\mathrm{up}}\cup G_{\mathrm{miss}-\mathrm{up}}$**.**

For this purpose, we exploit an N-step attenuation function, which can provide different proportion coefficients for the $n_{\mathrm{miss}}$ of different targets and is formulated as:

(41) $$\alpha_{P}(n_{\mathrm{miss}})=\left\{ \begin{array}{ll} \frac{1}{\exp\left((n_{\mathrm{miss}}-N_{W})/\lambda T\right)+1} & n_{\mathrm{miss}}\leq N_{W}\\ 0 & n_{\mathrm{miss}}>N_{W} \end{array}\right.$$

where $N_{W}$ is the size of the half-attenuation window, λ is a scaling factor that can be used to adjust the attenuation rate and T is the sampling period of sensor. Subsequently, the proportion coefficients of all undetected targets at each time step are normalized to decide the compensation proportion of the underlying weight from $W_{\mathrm{tr}}$ (Step 2 of Algorithm 1).

In view of the overall behavior of the spooky effect [17], the proposed method in Algorithm 1 can help to prevent the detected components from gaining extra weights that originate from the PHD of the undetected targets. Accordingly, the updated weights of the undetected targets are relatively concentrated in the predicted vicinity of the undetected targets, which are compensated according to the missed detection information $n_{\mathrm{miss}}$ in multiple frames. Thus, the PHD mass interaction via missed detections in the CPHD filter is reduced effectively. More importantly, following the argument in [18], the proposed method has no effect on the cardinality estimation because the global PHD mass remains unchanged in the weight redistribution scheme.

By combining the $v_{k}(x,0)$ resulting from (38) and the $v_{k}(x,1)$ (parameterized by $G_{\mathrm{up}}$) resulting from Algorithm 1, the finally updated posterior intensity $v_{k}(x)$ can be written as:

(42) $$v_{k}(x)=v_{k}(x,1)+v_{k}(x,0)=\sum_{\beta =0}^{1}\sum_{j=1}^{J_{k}}w_{k,\beta }^{j}N(x;m_{k,\beta }^{j},P_{k,\beta }^{j})$$

where $J_{k}$ is the number of updated Gaussian components.

**Remark** **1.** *When targets really disappear, the proposed method in Algorithm 1 will regard the related components as undetected components and perform compensation to their weights. Consequentially, the results can help the filter to reserve the target information, but may lead to a slower response to target disappearance. This is acceptable because the situation of missed detections and target disappearances are difficult to distinguish within a few time steps. For practical MTT problems, there always exists a trade-off between alleviating performance degradation caused by missed detection and providing an excellent performance for track continuity [3,24], and it is also not advisable to declare target death prematurely. Empirically, considering the inherent inertia of the CPHD filter in response to cardinality changes [12],* $2\leq N_{W}\leq 4$ *is suggested for the purpose of reducing the spooky effect and preventing the adverse influence on the response speed to target disappearance. Since our weight redistribution scheme works only within the half-attenuation window for a given undetected component, while the dynamic reweighting method [18] tends to persistently assign extra weight to such a component as long as the component exists, our method will possess certain advantages upon target disappearance, which is demonstrated by the simulations presented subsequently in this paper.*

### 3.2. Implementation Issues

Mixture component pruning: To keep a reasonable number of mixture components in the GM-CPHD recursion, some heuristic pruning and merging procedures are necessary, where the tag β and $n_{\mathrm{miss}}$ of the GM components should be considered. Based on the basic method proposed in [11], a modified pruning and merging procedure is given in Algorithm 2 to accommodate the proposed filter. In Algorithm 2, the notation $\beta^{j}$ is used to represent the tag β associated with the component whose index is j.

State extraction: The joint estimation of the time-varying number of targets and their individual states in the GM-CPHD filter involves first estimating the target number $N_{k}$ by a maximum a posterior (MAP) estimator [12] and then selecting the corresponding number of Gaussian components with the largest weights to report their means as the estimated multi-target state. When the estimated multi-target state ${\hat{X}}_{k}=\left\{m_{k}^{1},m_{k}^{2},\ldots ,m_{k}^{N_{k}}\right\}$ is obtained, the tag β=1 and $n_{\mathrm{miss}}=0$ need to be assigned to the confirmed components whose means are first reported as state estimates.

**Algorithm 2.** Pseudo-code for the pruning and merging method**Given**
${\left\{w_{k,\beta^{j}}^{j},m_{k,\beta^{j}}^{j},P_{k,\beta^{j}}^{j},n_{k,miss}^{j}\right\}}_{i=1}^{J_{k}}$, a truncation threshold $T_{\mathrm{trun}}$, a merging threshold $U_{\mathrm{merg}}$ and a maximum allowable number of Gaussian terms $J_{\max}$.**Step 0.** Set l=0 and $I=\text{\{}i=1,\ldots ,J_{k}|w_{k,\beta^{i}}^{i}>T_{\mathrm{trun}}\text{\}}$.**Step 1. repeat**      l=l+1.      $j=\arg\underset{i\in I}{\max}\;w_{k,\beta^{i}}^{i}$.      $L=\{i\in I|{\left(m_{k,\beta^{i}}^{i}-m_{k,\beta^{j}}^{j}\right)}^{T}{\left(P_{k,\beta^{j}}^{j}\right)}^{-1}(m_{k,\beta^{i}}^{i}-m_{k,\beta^{j}}^{j})\leq U_{\mathrm{merg}}\}$.      ${\tilde{\beta }}^{l}=\beta^{j}$, ${\tilde{n}}_{k,\mathrm{miss}}^{l}=n_{k,\mathrm{miss}}^{j}$.      ${\tilde{w}}_{k,{\tilde{\beta }}^{l}}^{l}=\sum_{i\in L}w_{k,\beta^{i}}^{i}$, ${\tilde{m}}_{k,{\tilde{\beta }}^{l}}^{l}=\frac{1}{{\tilde{w}}_{k,{\tilde{\beta }}^{l}}^{l}}\sum_{i\in L}w_{k,\beta^{i}}^{i}m_{k,\beta^{i}}^{i}$.      $P_{k,{\tilde{\beta }}^{l}}^{l}=\frac{1}{{\tilde{w}}_{k,{\tilde{\beta }}^{l}}^{l}}\sum_{i\in L}w_{k,\beta^{i}}^{i}\left(P_{k,\beta^{i}}^{i}+({\tilde{m}}_{k,{\tilde{\beta }}^{l}}^{l}-m_{k,\beta^{i}}^{i}){\left({\tilde{m}}_{k,{\tilde{\beta }}^{l}}^{l}-m_{k,\beta^{i}}^{i}\right)}^{T}\right)$.      I=I\L.    **Until**
I= ∅.    If $l>J_{\max}$, select the Gaussian terms ${\left\{{\tilde{w}}_{k,{\tilde{\beta }}^{i}}^{i},{\tilde{m}}_{k,{\tilde{\beta }}^{i}}^{i},{\tilde{P}}_{k,{\tilde{\beta }}^{i}}^{i},{\tilde{n}}_{k,\mathrm{miss}}^{i}\right\}}_{i=1}^{J_{\max}}$ with the largest weights and take $l=J_{\max}$.**Output:**
${\left\{{\tilde{w}}_{k,{\tilde{\beta }}^{i}}^{i},{\tilde{m}}_{k,{\tilde{\beta }}^{i}}^{i},{\tilde{P}}_{k,{\tilde{\beta }}^{i}}^{i},{\tilde{n}}_{k,\mathrm{miss}}^{i}\right\}}_{i=1}^{l}$**.**

### 3.3. Gating Strategy

In theory, the GM-CPHD filter has a computational complexity of order $O(n_{k}{\left|Z_{k}\right|}^{3})$ at each time step [13,23]. This can be very computationally demanding when a large number of clutter measurements is generated in the tracking scenarios because of the complicated backgrounds and the influence of noise. It has been demonstrated that the elliptical gating method [22] is effective to simplify the filtering update calculations via removing the potential clutter measurements, which can be formulated as:

(43) $$z_{k,m}\left\{ \begin{array}{ll} \in {\tilde{Z}}_{k} & \mathrm{if}\;\exists (m,j)|{\left[z_{k,m}-g(m_{k|k-1}^{j})\right]}^{T}S_{k|k-1}^{-1}{\left[z_{k,m}-g(m_{k|k-1}^{j})\right]}^{T}\leq T_{g}\\ \notin {\tilde{Z}}_{k} & \mathrm{otherwise} \end{array}\right.$$

where ${\tilde{Z}}_{k}$ is the set of the valid measurements and $T_{g}$ is the gate threshold. The value of $T_{g}$ depends on a given gate probability $P_{g}$ and the dimension of the measurement $n_{z}$ (see [22] for more details). The validation region is typically an ellipsoid whose center point is the predicted measurement of each component. On the basis of Equation (43), an adaptive gating method that directly utilizes the predicted weights of the Gaussian components to increase the gate thresholds was proposed in [23], where for a specified component indexed by j, the adaptive gate threshold is given by $T_{g}^{\prime }=T_{g}(1+w_{k|k-1}^{j})$. It is clear that the resulting gate thresholds are always larger than those of the elliptical gate, and some of them also tend to be excessively enlarged for the components with significant weights. This effect is not preferable owing to the drawback that more clutter measurements will be included and hence goes against the optimum solution (in the minimal volume sense for a given in-gate probability of target-originated measurements [25]). Based on the proposed N-step attenuation function, the following heuristic gating strategy is proposed to adjust the gate threshold:

(44) $${\hat{T}}_{g}=\left\{ \begin{array}{ll} \frac{T_{g}}{\alpha_{P}(n_{\mathrm{miss}}^{j})} & \beta^{j}=1,n_{\mathrm{miss}}^{j}\neq 0,n_{\mathrm{miss}}^{j}\leq N_{W}\\ T_{g} & \mathrm{otherwise} \end{array}\right.$$

where the influence of consecutive missed detection is considered for the confirmed GM components. The basic principle behind the proposed gating method is that the undetected targets in the previous time step will have a larger statistical error in their predicted measurements than those of the detected targets to some degree [24]. Therefore, we allow a large gate size, which can be adaptive in accordance with the degree of missed detection, for the confirmed component with missed detection, such that it can be more conservative in the region where the corresponding measurement may appear. Although the proposed gating strategy seems to be relatively conservative as compared with the adaptive gating method, it is expected to offer comparable robustness in the presence of detection uncertainty and clutter with a reasonable $T_{g}$.

## 4. Simulation

**Figure 1:**
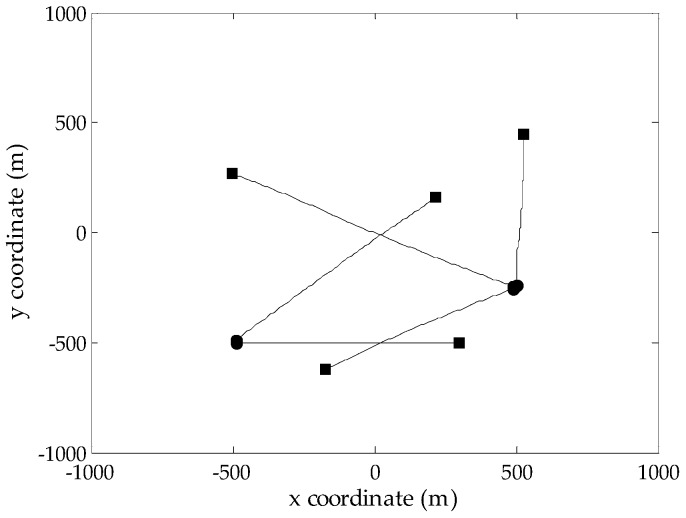
True target tracks of Scenario 1 in the *xy*-plane; the start/end points for each track are denoted by •/■, respectively.

**Figure 2:**
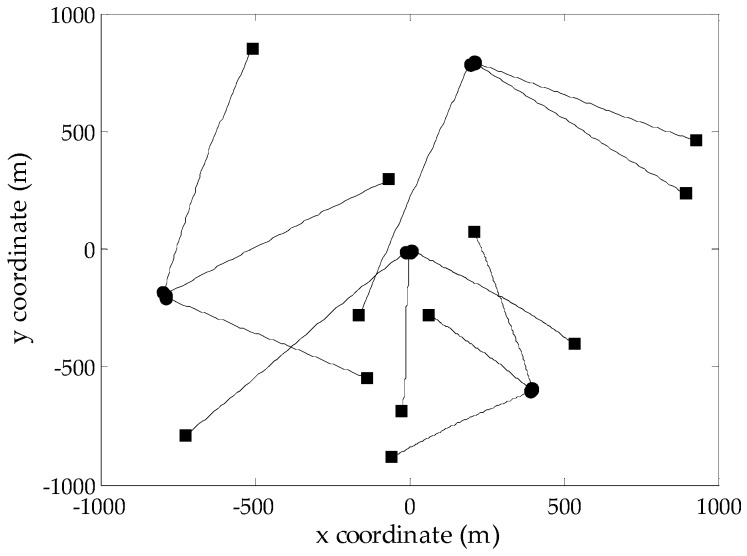
True target tracks of Scenario 2 in the *xy*-plane; the start/end points for each track are denoted by •/■, respectively.

To evaluate the performance of the proposed GM-CPHD filter, simulations are performed on two-dimensional scenarios generated according to [12], where the same target motion model and observation model are adopted, as well as the corresponding noise models. Considering that the number of targets in the scene has a significant effect on the spooky effect, two multi-target scenarios with a maximum of five targets and twelve targets, respectively, are designed over the surveillance region [−1000,1000]m×[−1000,1000]m. Due to the existence of target births and deaths, the number of targets in both scenarios is time varying. The probability of target survival is $p_{S,k}=0.99$. As done in [12], the target birth model follows a Poisson RFS, whose intensity is a priori known. Clutter is uniformly distributed in the field of view, which is modeled as a Poisson RFS with the average clutter intensity $\lambda_{c}=0.25\times 10^{-5}\;m^{-2}$ (the average number of clutters is 10 at each scan). Figure 1 and Figure 2 show the true target tracks in Scenarios 1 and 2, respectively, along with the start and stop positions of each track. Note that there are two targets whose tracks cross at Time Step 50 in Scenario 1. The optimal sub-pattern assignment (OSPA) distance [26] is used to measure the performance of different multi-target filters. According to the analysis presented in [26], the metric values are generated using the OSPA parameters p=2 and c=100.

### 4.1. Evaluation of Different CPHD Filters

In this part, we compare the performance of the proposed GM-CPHD filter without gating, the P-GM-CPHD filter, with those of the standard GM-CPHD filter [12] and the dynamic reweighting GM-CPHD filter (DR-GM-CPHD) [18] via Monte Carlo (MC) simulations. Assuming that the targets are measured by a sensor with sampling period T=1 s, the parameters $T_{\mathrm{trun}}=1\times 10^{-5}$, $J_{\max}=100$ and $U_{\mathrm{merg}}=4$ are used in all of these filters [12]. In addition, the detection threshold $T_{\det}=0.2$ is used in our method, and the sizes of the time fading window and scaling factor are $N_{W}=3$ and λ=0.8, respectively. 

**Figure 3:**
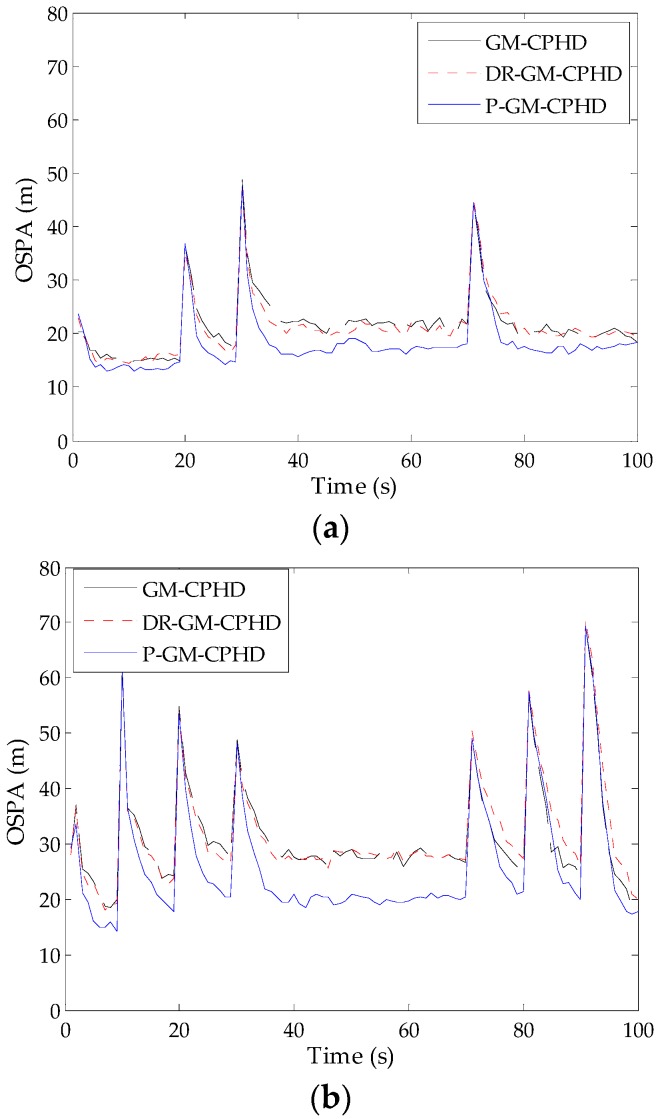
Optimal sub-pattern assignment (OSPA) distance versus time for the three filters: (**a**) the results obtained from Scenario 1; (**b**) the results obtained from Scenario 2. P, proposed; DR, dynamic reweighting.

To verify the performance of the P-GM-CPHD filter, 500 Monte Carlo (MC) runs are performed on the fixed target tracks presented in Scenarios 1 and 2, but with independently generated clutter and measurements for each trial. For comparison, the corresponding simulations are also performed using the standard GM-CPHD filter and DR-GM-CPHD filter. Figure 3 gives the statistical results of the OSPA distance for different filters, where the detection probability $p_{D,k}=0.90$ and clutter intensity $\lambda_{c}=0.25\times 10^{-5}\;m^{-2}$ are used during these simulations. It can be seen that the P-GM-CPHD filter outperforms the GM-CPHD filter and DR-GM-CPHD filter on the overall miss distance. The lower OSPA distance indicates high estimation accuracy in terms of both cardinality and multi-target state. The improved estimation performance is mainly attributed to the effective inhibition of the spooky effect, which is unfavorable for detecting new targets (new targets appear at Time Steps 1, 20 and 30 in Scenario 1 and at Time Steps 1, 10, 20 and 30 in Scenario 2) and maintaining the tracks of the existing targets. A closer examination of the results in Figure 3 reveals that the proposed weight redistribution scheme only has a slight influence on the filter with respect to the response speed to target disappearance (targets disappear at Time Step 70 in Scenario 1, and at Time Steps 70, 80 and 90 in Scenario 2), and the resulting OSPA distance around the time steps of target disappearance is very close to that of the standard GM-CPHD filter. By contrast, the dynamic reweighting method in [18] performs worse than our method and the GM-CPHD filter. As said before, the reason is that the dynamic reweighting method always assigns the excess weights to the components associated with the disappeared targets at each time step unless such components are removed by the pruning procedure.

**Figure 4:**
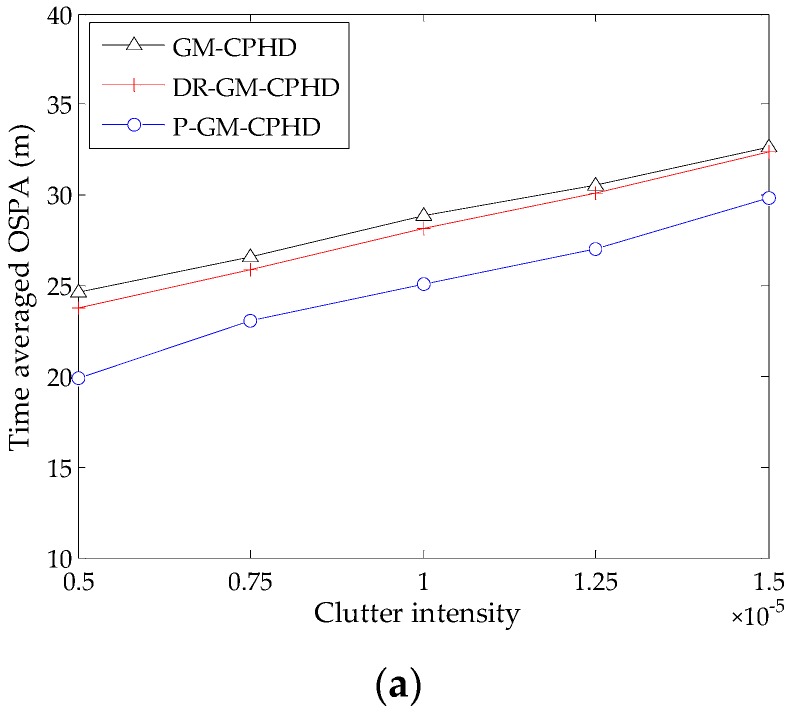

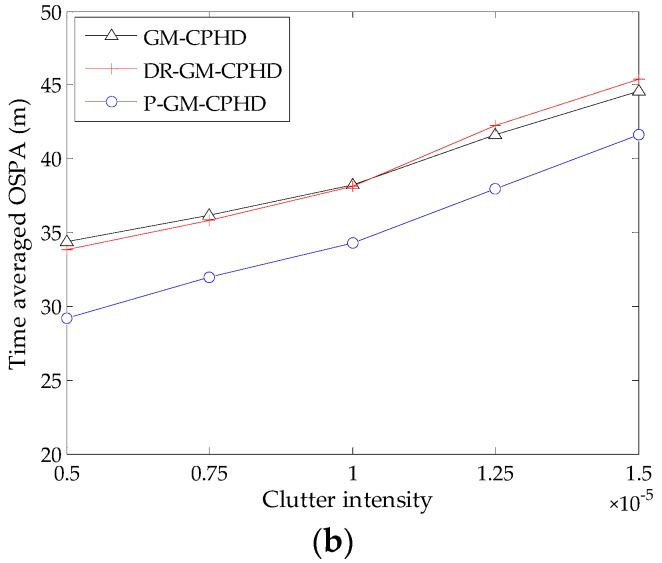
OSPA distance versus varying clutter intensity for the three filters ($p_{D,k}=0.90$): (**a**) the results obtained from Scenario 1; (**b**) the results obtained from Scenario 2.

In addition, we also investigate the performance of the filters under consideration by increasing the clutter intensity to $\lambda_{c}$; the resulting time-averaged OSPA distances against clutter intensities are shown in Figure 4. As expected, the OSPA distance increases with higher clutter intensities. Moreover, the results show that the DR-GM-CPHD filter exhibits unreliability when dealing with the scenarios where there exist heavy clutter and a large number of targets, while the P-GM-CPHD filter still yields the best performance in terms of estimation accuracy and robustness. This means that our method has high reliability for multi-target tracking in the presence of clutter and detection uncertainty.

**Figure 5:**
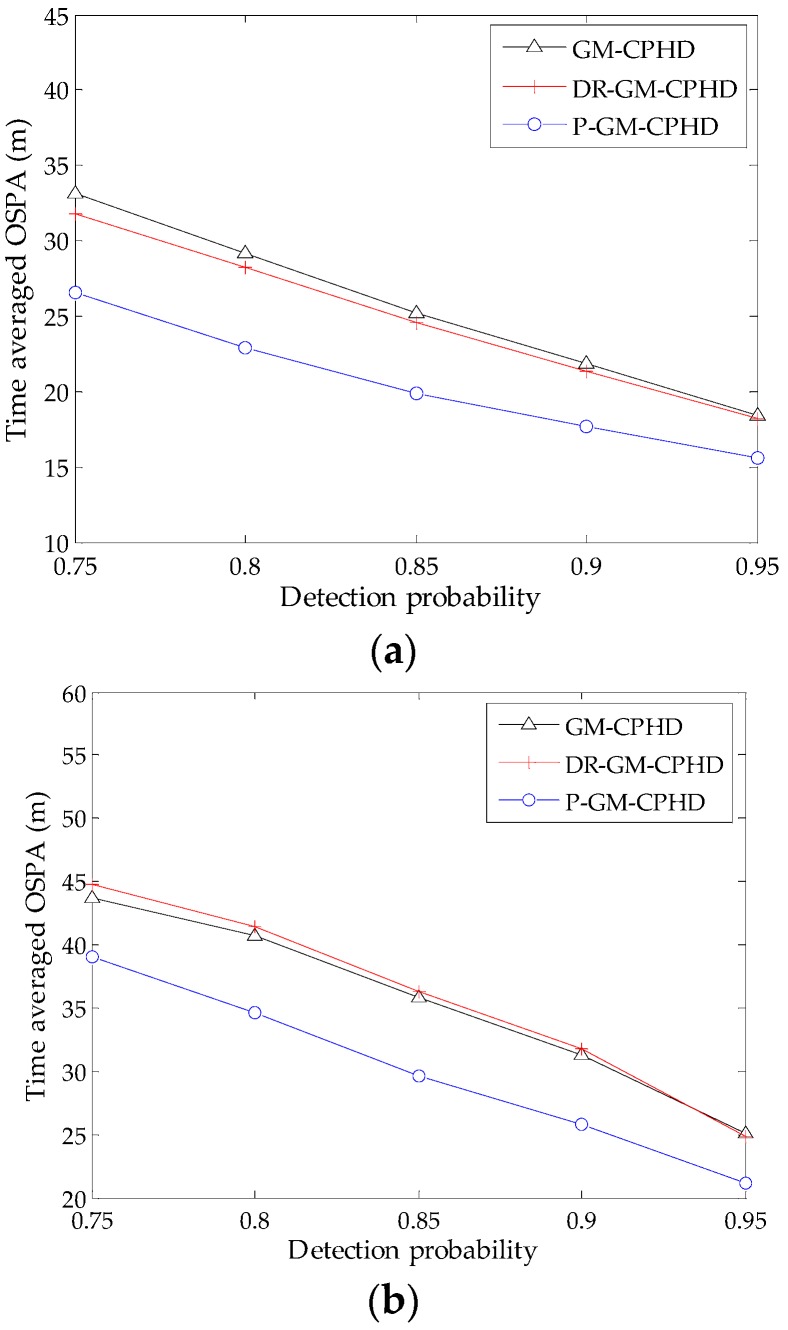
Average OSPA distance versus varying detection probability for the three filters ($\lambda_{c}=0.25\times 10^{-5}\;m^{-2}$): (**a**) the results obtained from Scenario 1; (**b**) the results obtained from Scenario 2.

To investigate the influence of missed detection, the tracking performances of the three filters are examined against different detection probabilities under Scenarios 1 and 2 via 500 MC simulations. The resulting time-averaged OSPA distances versus various probabilities of detection under the condition of fixed $\lambda_{c}$ are shown in Figure 5. Generally, the low probability of detection leads to an increase in OSPA distance. However, it can be seen from Figure 5 that the performance improvement by using the DR-GM-CPHD filter is limited for most cases, and the filter even causes performance degradation under Scenario 2 as compared with the standard GM-CPHD filter. By contrast, the superiority of the P-GM-CPHD filter is remarkable, which exhibits the best reliability among these methods. Thus, it can be said that the proposed method is more effective in dealing with the spooky effect.

### 4.2. Evaluation of Different Gating Methods

**Figure 6:**
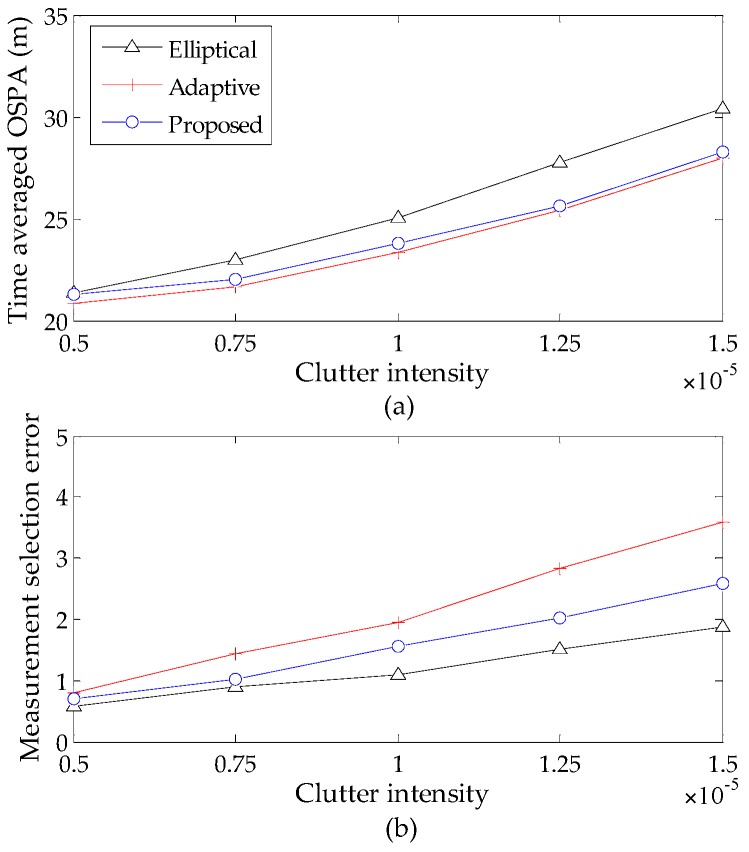
Tracking performance versus varying clutter intensity ($p_{D,k}=0.90$): (**a**) time averaged OSPA distance versus varying clutter intensity; (**b**) measurement selection error versus varying clutter intensity.

**Figure 7:**
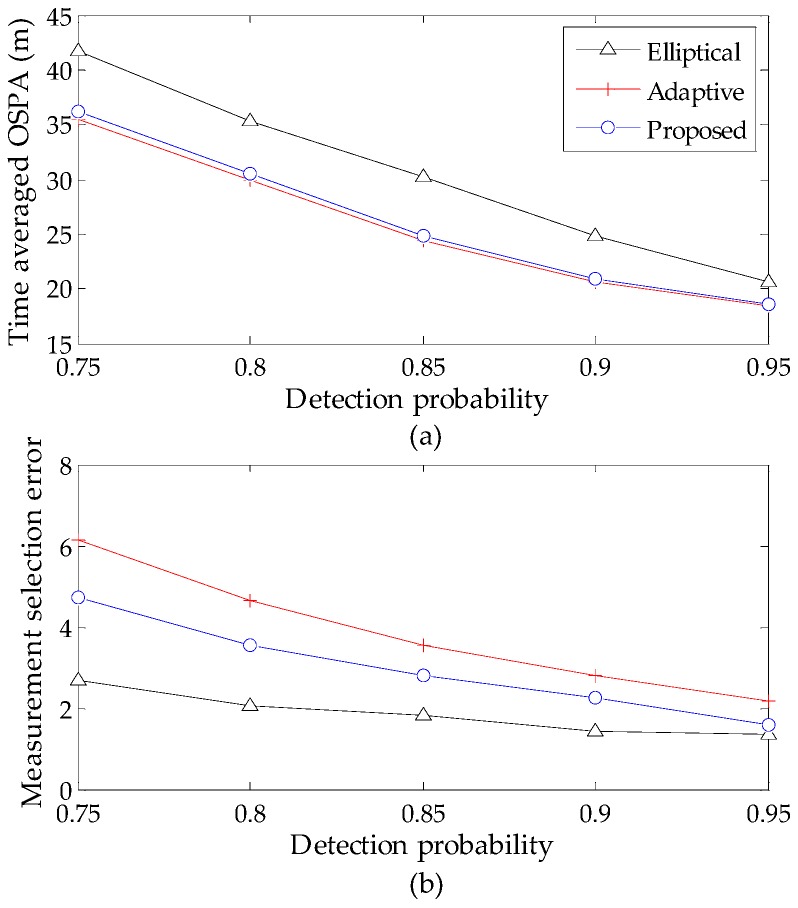
Tracking performance versus varying detection probability ($\lambda_{c}=0.25\times 10^{-5}\;m^{-2}$): (**a**) time averaged OSPA distance versus varying detection probability; (**b**) measurement selection error versus varying detection probability.

To compare the performance of the elliptical gating method [22], the adaptive gating method [23] and the proposed gating method for the GM-CPHD filter, a comprehensive evaluation is performed using the P-GM-CPHD filter, but with different gating methods for varying clutter intensities and detection probabilities. The measurement selection error, defined as the difference between the number of the true measurements, that of the selected measurements at each time step and the time-averaged OSPA distance are taken as the metrics. The time-averaged OSPA distance and measurement selection error versus the clutter intensity are shown in Figure 6. The time-averaged OSPA distance and measurement selection error versus the detection probability are shown in Figure 7. Note that all of these results are obtained by using Scenario 1, and the basic gate probability is set to $P_{g}=1-10^{-4}$ [22] for the implementation of the three gating methods.

It is shown that the high clutter rates and low probability of detections will cause performance degradation, and this phenomenon becomes increasingly evident with the increase of detection uncertainty. The results in Figure 6 and Figure 7 demonstrate that the proposed gating method gives a better performance, which is very similar to the adaptive gating method. It is known that the gate sizes of the latter are always larger than the standard elliptical gating method and our method to ensure the inclusion of true target-originated measurements. Accordingly, the improper enlargement of the gate size will allow more measurements to be selected and, hence, result in large measurement selection error. This in turn limits the improved performance in computational efficiency. By contrast, the proposed gating strategy makes it possible to achieve a favorable result between the ability of selecting true measurements and that of shielding clutter measurements without deteriorating the filter performance. In addition, to present an intuitive indication of the improvement in computational efficiency, the average processing time (corresponding to one iteration in MATLAB implementation) of the P-GM-CPHD filter and that of the proposed gating P-GM-CPHD filter are given in Table 1.

## 5. Conclusions

This paper proposed an improved GM-CPHD filter, which aims at addressing the spooky effect in the original filter for MTT. More specifically, we proposed a weight redistribution scheme for the filter, which provides an ability to keep the concentration of PHD mass for the undetected targets based on the information in multiple frames. Besides, by exploiting the information of missed detections recorded during the filtering process, an efficient gating strategy that can adaptively enlarge the gate sizes was also proposed to alleviate the computational burden of the proposed filter in cluttered scenarios. Simulations verified that the proposed filter can achieve significant improvements in estimation accuracy and robustness to detection uncertainty, thereby implying an enhanced tracking performance. Moreover, the proposed gating method also showed some advantages over the existing solutions. As future work, extending the proposed filter to a multiple passive sensors system will be an important topic.

## Figures and Tables

**Table 1:** Average processing times of the proposed filters (s).

Average Clutter Intensity	Processing Time (Gating)	Processing Time (No Gating)
$0.75\times 10^{-5}\;m^{-2}$	2.30	3.18
$1.00\times 10^{-5}\;m^{-2}$	2.65	4.87
$1.25\times 10^{-5}\;m^{-2}$	3.22	8.35
